# Assessment of longitudinal beam property and contrast uniformity for 256‐ and 320‐row area detector computed tomography scanners in the 160‐mm nonhelical volume‐acquisition mode

**DOI:** 10.1002/acm2.12670

**Published:** 2019-06-29

**Authors:** Takanori Hara, Shinji Niwa, Atsushi Urikura, Kosuke Matsubara, Takashi Hoshino, Eiji Nishimaru, Takuya Taniguchi

**Affiliations:** ^1^ Department of Medical Technology Nakatsugawa Municipal General Hospital Nakatsugawa Gifu Japan; ^2^ Department of Diagnostic Radiology Shizuoka Cancer Centre Nagaizumi Shizuoka Japan; ^3^ Institute of Medical, Pharmaceutical and Health Sciences Kanazawa University Kanazawa Ishikawa Japan; ^4^ Department of Radiological Technology Osaka College of High Technology Osaka‐shi Osaka Japan; ^5^ Department of Radiology Hiroshima University Hospital Minami‐ku Hiroshima Japan; ^6^ Department of Radiology Asahi University Hospital Hashimoto‐cho Gifu Japan

**Keywords:** 160‐mm x‐ray beam, area detector computed tomography (ADCT), half‐value layer (HVL), heel effect, radiation dose, volume‐acquisition mode

## Abstract

**Background:**

Because the x‐ray property of patient longitudinal axis in area detector computed tomography (ADCT) depends on a heel effect, radiation dose and beam quality are not uniform along the long axis of the patient.

**Objective:**

This study aimed to measure the longitudinal beam properties and contrast uniformity of ADCT scanners in the 160‐mm nonhelical volume‐acquisition (NVA) mode and provide useful datasets for the radiation dose reduction in ADCT examinations.

**Materials and Methods:**

Two different types of ADCT scanners were used in this study. To assess the heel effect in 256‐ and 320‐row ADCT scanners, we measured dose profile, half‐value layer, and iodine contrast uniformity along longitudinal beam direction.

**Results:**

The maximum effective energy difference within a 160‐mm x‐ray beam is approximately 4 keV. Maximum radiation dose on the anode side of the x‐ray tube showed approximately 40%–45% reduction compared with that on the isocenter position; the heel effect properties longitudinally differed throughout the x‐ray beam, and the decrease in the radiation dose in 256‐ and 320‐row ADCT scanners was observed on the patient table side and gantry side respectively. The CT numbers of iodinated solutions for 256‐row ADCT scanner were independent of the heel effect; nevertheless, the CT numbers of 320‐row ADCT scanner tended to increase on the patient table (cathode) side.

**Conclusion:**

This study reveals that the radiation dose on the anode side of the x‐ray tube shows approximately 40%–45% reduction compared with that on the isocenter position, and the heel effect properties for 256‐ and 320‐row ADCT scanners longitudinally differ throughout the x‐ray beam. The x‐ray tube for individual ADCT scanners is mounted in an opposite direction along the long axis of the patient.

## 1 INTRODUCTION

The recently introduced area detector computed tomography (ADCT) scanner has multiple acquisition modes,^1^, ^2^, ^3^, ^4^, ^5^ including a nonhelical volume‐acquisition (NVA) mode. This mode, which utilizes a 160‐mm‐wide x‐ray beam, is particularly useful in infants and small children for reducing the frequency of motion‐related artifacts and the need for sedation. Compared with the helical acquisition mode, the NVA mode reduces the radiation dose owing to the lack of Z‐axis overranging and decreased overbeaming effects.^5^, ^6^ Modern ADCT scanners are equipped with features for modulation of radiation dose (tube current), such as automatic exposure control (AEC)^7^, ^8^ and organ dose modulation (ODM).^9^, ^10^ These techniques may be used to optimize radiation dose administered to patients. The Z‐axis (longitudinal) tube current modulation of AEC can be performed only in the helical acquisition mode; however, the 160‐mm NVA mode cannot dynamically adjust the longitudinal radiation dose per one rotation.

Although the available evidence on the side effects of low dose radiation still remains a matter of discussion, it is generally believed that there is a linear‐no threshold risk relationship; in addition, rapidly dividing cells are more sensitive to radiation; therefore, the risk of cancer from radiation exposure is higher in children compared to adults, with infants at the greatest risk^11^; radiologists should consider minimizing the radiation dose administered to children.

The use of a wide x‐ray beam fosters the angular dependence of the emitted spectrum of the x‐ray tube, which is known as the heel effect; it is a systematic effect that is caused by the geometry of a rotating anode x‐ray tube and known to cause inhomogeneous dose distributions.^12^, ^13^ We inferred from this effect that an organ‐absorbed dose was not constant along the long axis of the patient in the 160‐mm NVA mode; furthermore, the effect is not managed in the clinical practice with the ADCT scanners.^5^, ^14^

To the best of our knowledge, the longitudinal properties of the 160‐mm x‐ray beam for 256‐ and 320‐row ADCT scanners have not been previously compared. Furthermore, the placement of the anode (or the cathode) of the x‐ray tube in the gantry is not sufficiently provided in the specifications of the individual scanner.^15^, ^16^, ^17^ Therefore, if the x‐ray beam properties of respective ADCT scanners are revealed, we can reduce the radiation dose administered to the critical organs, such as eye lens,^18^ during ADCT scan in the 160‐mm NVA mode by applying the properties of the heel effect. In this study, we measured the longitudinal beam properties and contrast uniformity of 256‐ and 320‐row ADCT scanners in the 160‐mm NVA mode and provided useful datasets for the radiation dose reduction in ADCT examinations.

## 2 MATERIALS AND METHODS

### 2.1 ADCT scanners

The following ADCT scanners were used in this study: Revolution CT (GE Medical Systems, Milwaukee WI, USA) and Aquilion ONE ViSION Edition (Canon Medical Systems, Tokyo, Japan). The Revolution CT scanner is equipped with 256 detector rows (256‐row ADCT), and the Aquilion ONE ViSION Edition scanner is equipped with 320 detector rows (320‐row ADCT). Both scanners can achieve a detector coverage of 160 mm in a single rotation at the isocenter of rotation (detector configuration: 256 × 0.625 mm for 256‐row ADCT; 320 × 0.5 mm for 320‐row ADCT).

### 2.2 Dosimeter systems

The following dosimeter systems were used in this study: Piranha 657 (RTI Piranha; RTI Electronics, Mölndal, Sweden) dosimeter for dose profile and half‐value layer (HVL) measurements^19^; CT Dose Profiler (RTI CTDP; RTI Electronics, Mölndal, Sweden) probe for dose profile measurement.^20^ The RTI Piranha dosimeter is a multipurpose x‐ray output analyzer with a 3‐ × 21‐mm x‐ray detection area. This dosimeter has an optimized filter packages for five different kV ranges, and can automatically measure the output such as air kerma for various modalities. This dosimeter was previously calibrated at RTI laboratory in Sweden. The RTI CTDP probe is a point dose detector with a 250‐μm solid‐state sensor placed 30 mm from the pointed end of the probe. For dose profile measurement in cone beam CT, the RTI CTDP probe uses the RTI Mover (RTI Electronics, Mölndal, Sweden)^21^ which is the dedicated drive motor system. The RTI CTDP probe and RTI Mover are connected via thin wire. The RTI Mover can measure the dose profile by continuously moving a 250‐μm thin sensor that is implanted into the RTI CTDP probe along the longitudinal x‐ray beam. All data were transferred to a laptop via bluetooth and analyzed using Ocean software (RTI Electronics, Mölndal, Sweden).

### 2.3 Measurement of longitudinal beam property

#### 2.3.1 Dose profile

**Figure 1:**
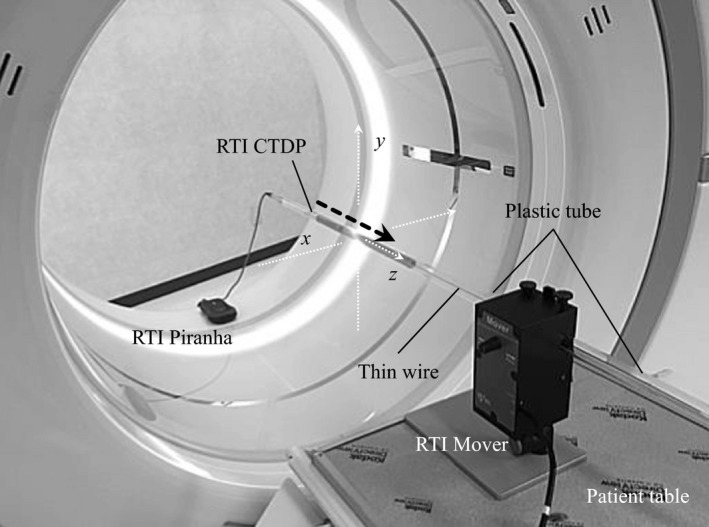
Photograph of the experimental setup for dose profile measurements. The small sensor embedded in the computed tomography dose profiler probe was moved at a constant speed of 83 mm/s by the RTI Mover during continuous use of the 160‐mm non‐helical volume‐acquisition mode.

Figure 1 shows the experimental setup for dose profile measurements in the ADCT scanner. Using a position setting button on the gantry and a flexible ball joint on the RTI Mover, the RTI CTDP probe was placed free‐in‐air at the outside position of 160‐mm beam along the Z‐axis. For dose profile measurement, we moved the CTDP probe using the RTI Mover along the Z‐axis with a constant speed of 83 mm/s during continuous use of the 160‐mm NVA mode. The dose profiles were assessed in the 160‐mm NVA mode in the respective ADCT scanners. To improve the accuracy of measurement, these values were calculated from three consecutive measurements. Scans were performed with tube voltage: 120 kVp; tube current: 200 mA; rotation time: 1.0 s, beam width: 160 mm, bowtie filter type: small; and scan type: continuous mode. The displayed volume computed tomography dose index (CTDI_vol_) per one rotation for respective CT scanners were as follows: for 256‐row ADCT, 28.2 mGy (16‐cm phantom) and for 320‐row ADCT, 39.8 mGy (16‐cm phantom).

#### 2.3.2 Beam quality

Effective energy of a poly‐energetic x‐ray beam is defined as energy equivalent to that of a mono‐energetic photon beam, which yields the same HVL, and it is closely related to the organ‐absorbed dose.^22^ HVL is generally used as a practical value for assessing x‐ray beam quality in the diagnostic field and can be measured using a high‐purity aluminum filter and a dedicated ionization chamber.^23^

**Figure 2:**
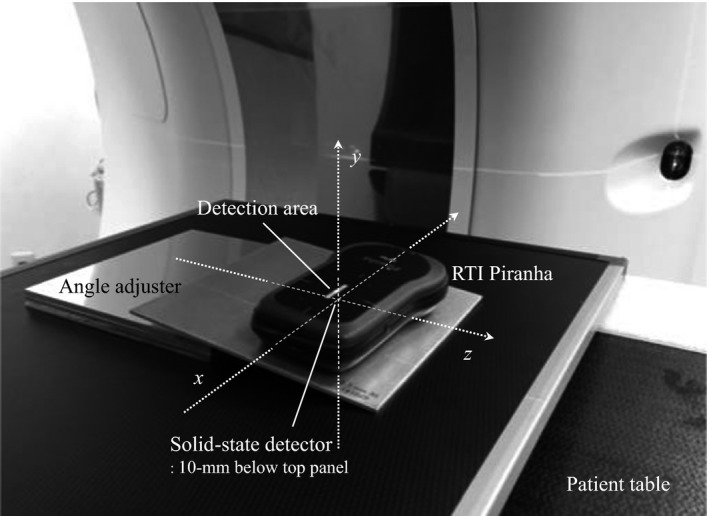
Photograph of the experimental setup for half‐value layer measurements. The slant angles of the detection area plane in the RTI Piranha dosimeter at the respective locations were adjusted by using digital clinometer.

Figure 2 shows the experimental setup for HVL measurements in the ADCT scanner. Using a position setting button on the gantry, the RTI Piranha dosimeter was placed at the isocenter position (0 mm) and the off‐center position along longitudinal beams of ± 30, ±60, and ± 75 mm. Moreover, to obtain an accurate vertical incidence of the x‐ray to the detection area plane of the dosimeter at the respective locations, we calculated the slant angles of the detection area plane at the respective longitudinal beam locations based on DICOM tag information and few previous reports.^1^, ^24^, ^25^ As a result of calculation, the slant angles of the detection area plane in the RTI Piranha dosimeter were as follows: 256‐row ADCT, 2.7° (±30 mm), 5.5° (±60 mm), and 6.8° (±75 mm); 320‐row ADCT, 2.9° (±30 mm), 5.7° (±60 mm), and 7.1° (±75 mm). These values were adjusted for an accuracy within ±0.1° by using a digital clinometer. The dosimeter can automatically detect whether the detector area is completely uniformly radiated by means of the “Position Check” function^19^ in the Ocean software program. Therefore, we performed “Position Check” test at the angle of respective locations. Moreover, to improve the accuracy of HVLs measurements, these values were calculated from three consecutive measurements; and finally, the effective energy along longitudinal beam locations was estimated from the HVL and linear attenuation coefficient of aluminium.^26^ Scans were performed with tube voltage: 80 and 120 kVp; tube current: 200 mA, exposure time: 1.0 s; beam width: 160 mm; bowtie filter type: small; and scan type: nonrotating exposure (stationary) mode with the x‐ray tube fixed at the 12‐o'clock position on the gantry.

In addition, to verify the accuracy of the RTI Piranha method for HVL measurement, we compared the HVL results obtained using the RTI Piranha method with those obtained using the conventional method.^27^ The conventional method was performed using a pencil ionization chamber (Victoreen Model 6000‐200; Fluke, Cleveland, OH, USA) and an electrometer (Victoreen Model 8000 NERO mAx). The ionizing dosimeter was previously calibrated at a laboratory accredited by the Japan Quality Assurance Organization. Scans were obtained with 256‐row ADCT scanner; tube voltage: 80, 100, and 120 kVp; tube current: 100 mA; exposure time: 1.0 s; bowtie filter type: medium; and scan type: stationary mode with 160‐mm wide x‐ray beam. To improve the accuracy of HVLs measurements, these values were calculated from three consecutive measurements.

### 2.4 Measurement of longitudinal contrast enhancement

Iodine is generally used as the contrast agent in contrast enhanced CT to better visualize vessels and organs. The iodine contrast enhancement (CT number) is closely related to the energy; the uniformity of CT numbers within the x‐ray beam is important for the diagnostic accuracy. However, the use of a wide x‐ray beam fosters the angular dependence of the emitted spectrum of the x‐ray beam (the heel effect) and causes inhomogeneous energy distributions along the long axis of the patient in CT.

**Figure 3:**
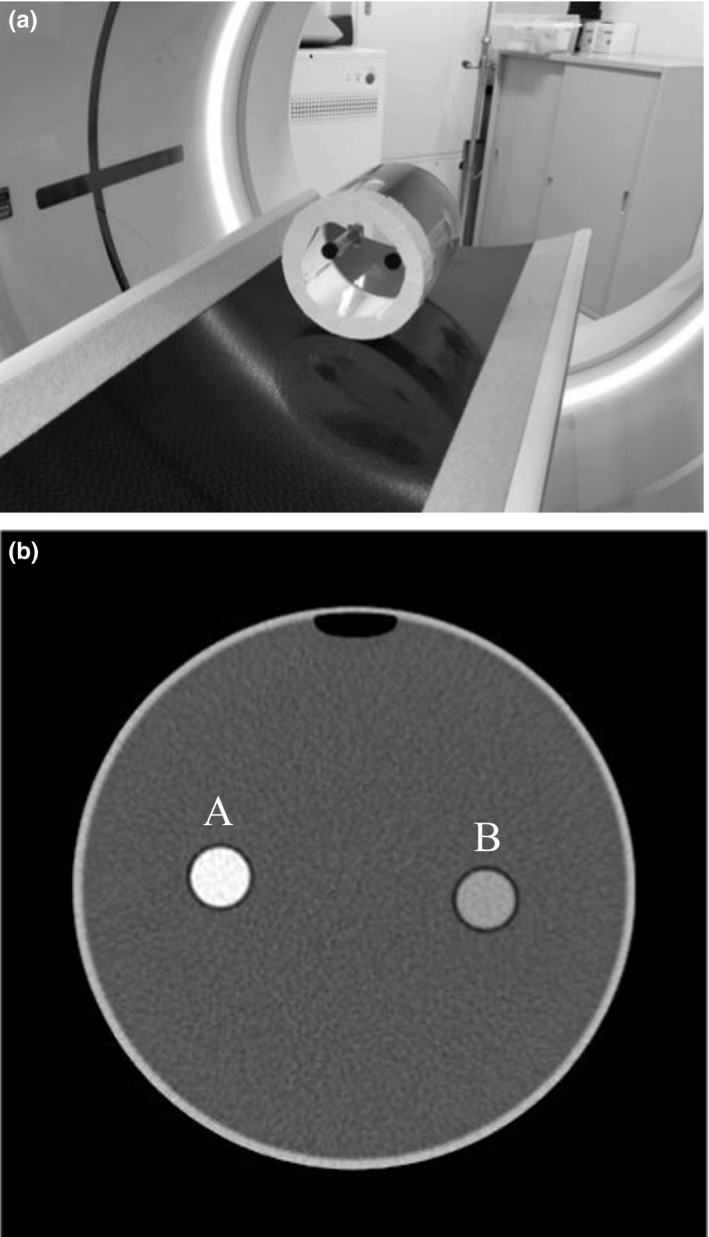
(a) Photograph of the phantom used for longitudinal contrast uniformity assessment. (b) Cross‐section image of the phantom. Iodinated solution of cylinder phantom was adjusted to (A) 7.3 and (B) 2.4 mgI/ml.

To assess the effects of the 160‐mm NVA mode on contrast enhanced head CT, we measured the longitudinal iodine contrast enhancement uniformity of CT numbers in individual ADCT scanners. A phantom was constructed using two plastic‐circular cylinders with 2.4 and 7.3 mgI/ml iodinated solutions, which were implanted into a 150‐mm cylindrical water bath phantom (Fig. 3). Scans were performed with tube voltage: 80 kVp; tube current: 200 mA; rotation time: 1.0 s; beam width: 160 mm; slice thickness: 5.0 mm; bowtie filter type: small; scan type: the 160‐mm NVA mode; display field‐of‐view: 200 mm; and a standard reconstruction kernel. The displayed CTDI_vol_ for respective CT scanners were as follows: for 256‐row ADCT, 10.2 mGy (16‐cm phantom) and for 320‐row ADCT, 14.2 mGy (16‐cm phantom). Acquired image data were obtained in the DICOM format and all the images were analyzed using the ImageJ software (ver. 1.46r; National Institutes of Health, Bethesda, MD, USA). The mean CT number of the respective iodinated solutions was obtained from 10‐mm diameter circular region of interest, which was set at the center of the cylinder phantom in the respective reconstructed images.

## 3 RESULTS

**Figure 4:**
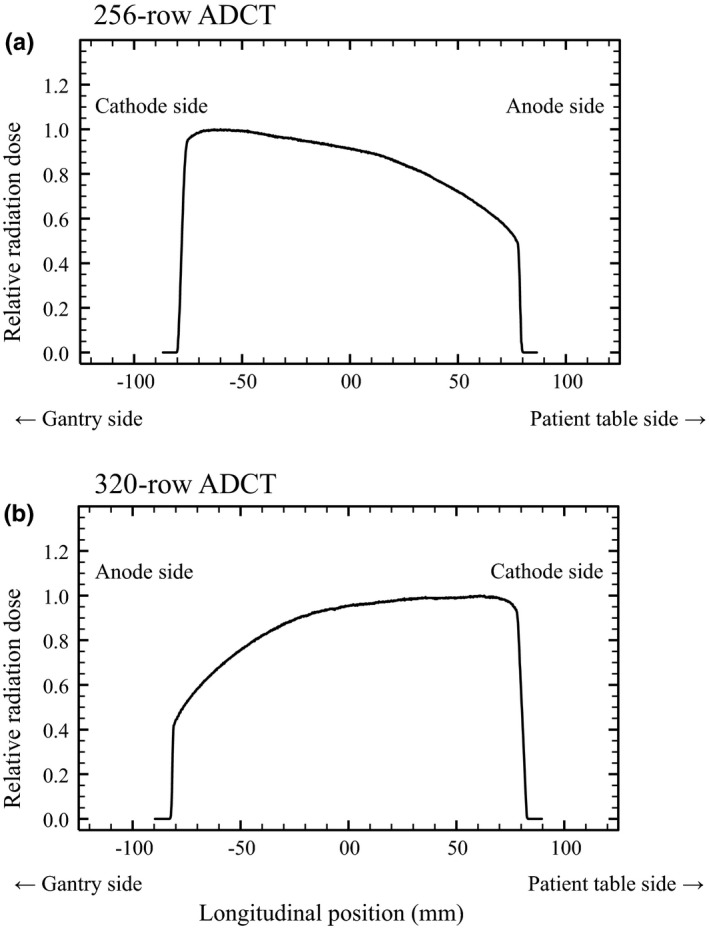
Dose profile results for (a) 256‐ and (b) 320‐row area detector computed tomography (ADCT) scanner. The heel effect in the respective ADCT scanners longitudinally differed along the beam.

The dose profiles measured along the longitudinal beam locations of 256‐row ADCT scanner are shown in Fig. 4(a). Dose distribution within the 160‐mm beam width indicated that dose administered to the area surrounding the −60‐mm off‐center position was approximately 9.5% greater than that administered to the area surrounding the isocenter position. The 75‐mm off‐center position from patient table side showed a 40% decrease in dose distribution compared with the isocenter position. Thus, in 256‐row ADCT scanner, the anode side of x‐ray tube is located on the patient table side. The measured dose profile along the longitudinal beam locations of 320‐row ADCT scanner are shown in Fig. 4(b). The dose distributions within the x‐ray beam width indicated that the area surrounding the 60‐mm off‐center position had an increase of approximately 5% compared with the isocenter position, whereas that around the −75‐mm off‐center position at gantry side had a decrease of approximately 45% compared with the isocenter position; thus, in 320‐row ADCT scanner, the anode side of x‐ray tube is located on the gantry side. The dose distribution along the longitudinal beam locations for 320‐row ADCT scanner had properties opposite to those of 256‐row ADCT scanner.

Table 1 shows the accuracy of HLV measurements in 256‐row ADCT scanner obtained using the RTI Piranha method. The HVL difference in the RTI Piranha dosimeter method and conventional method was <5% in all tube voltage settings. The maximum error values were −3.5% at a tube voltage of 120 kVp.

**Table 1:** Accuracy of half‐value layer with two different measurement methods.

Tube voltage (kVp)	Half‐value layer (mm Al)		Percent‐difference (%)
	RTI Piranha	Conventional	
80	4.11 ± 0.00	4.17 ± 0.01	−1.4
100	5.18 ± 0.00	5.34 ± 0.01	−3.0
120	6.11 ± 0.00	6.33 ± 0.00	−3.5

Note:

Data express mean ± standard deviation.

Table 2 shows the results of the effective energy along longitudinal beam locations. The effective energy in the range of −75 to 75 mm position for respective CT scanners were as follows: for 256‐row ADCT, 37.4–40.8 keV at a tube voltage of 80 kVp and 50.1–53.5 keV at a tube voltage of 120 kVp; for 320‐row ADCT, 41.6–37.4 keV at a tube voltage of 80 kVp and 53.1–48.5 keV at a tube voltage of 120 kVp. The longitudinal effective energy difference in 320‐row ADCT scanner was slightly larger than that in 256‐row ADCT scanner.

**Table 2:** Effective energy difference within 160‐mm x‐ray beam.

Tube voltage (kVp)	Position (mm)	Effective energy (keV)	
		256‐row ADCT	320‐row ADCT
80	−75	37.4	41.6
	−60	37.5	40.4
	−30	37.8	38.9
	0	38.3	38.1
	30	38.9	37.7
	60	40.0	37.4
	75	40.8	37.4
120	−75	50.1	53.1
	−60	50.2	51.8
	−30	50.5	50.2
	0	51.0	49.3
	30	51.6	48.8
	60	52.7	48.5
	75	53.5	48.5

**Figure 5:**
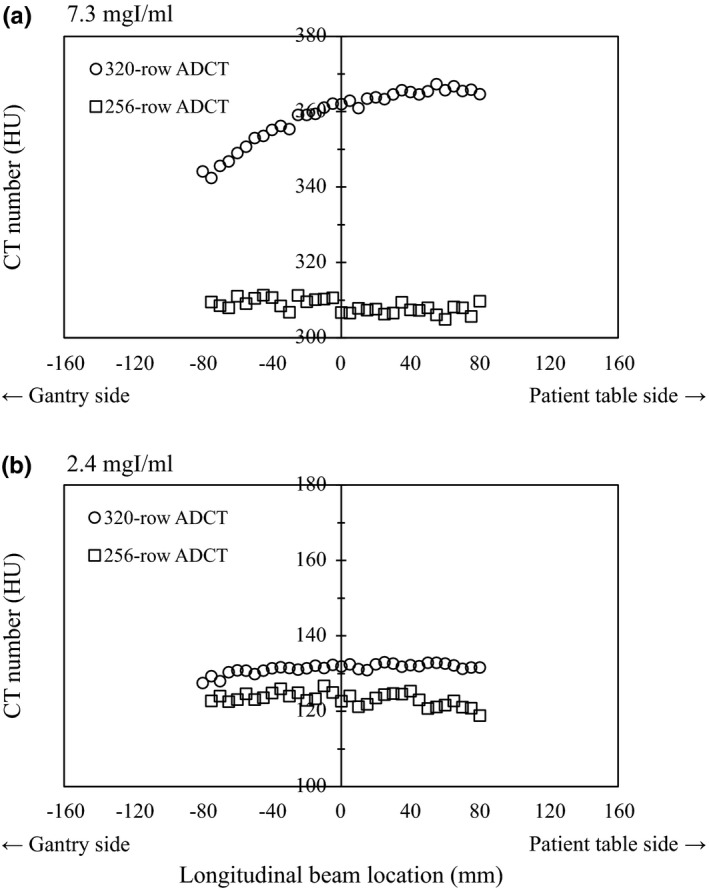
Influence of longitudinal beam locations on the computed tomography (CT) number of diluted contrast material. Mean CT numbers at the isocenter position (0 mm) were approximately as follows: for 256‐row area detector computed tomography (ADCT): 120 HU at 2.4 mgI/ml and 310 HU at 7.3 mgI/ml; for 320‐row ADCT: 130 HU at 2.4 mgI/ml and 360 HU at 7.3 mgI/ml.

The longitudinal contrast enhancement for individual ADCT scanners is shown in Fig. 5. The CT numbers of 320‐row ADCT scanner increased on the patient table side. Alternatively, the CT numbers of 256‐row ADCT scanner were almost equal along longitudinal beam locations, and were independent of the heel effect as observed for 320‐row ADCT scanner.

## 4 DISCUSSION

Recent reviews report that lifetime cancer risk estimates for those exposed as children were uncertain and might be a factor of two–three times higher than estimates for a population exposed at all ages^11^, ^28^; therefore, radiologists need to consider new approaches to decrease radiation risk.

The study results clearly demonstrated that the radiation dose on the anode side of the x‐ray tube showed a decrease of approximately 40%–45% compared with that on the isocenter position in the respective ADCT scanners with 160‐mm x‐ray beams. Moreover, heel effect properties differed longitudinally throughout the x‐ray beam. The decrease in the radiation dose in 256‐ and 320‐row ADCT scanners was observed on the patient table side and gantry side respectively. Therefore, we deduced that the x‐ray tube for 256‐ and 320‐row ADCT scanners is mounted in an opposite direction along the long axis of the patient, respectively, and the radiation dose on the anode side of the x‐ray tube is less than the isocenter position. In clinical practice, to reduce the radiation dose administered to the critical organs, such as eye lens, during ADCT scan in the 160‐mm NVA mode, radiologists might be better to keep the patient oriented such that the anode side of the x‐ray tube faces the critical organ side. In addition, as gantry tilting technique can keep the critical organ just out of the path of the x‐ray beam with high accuracy, we can accordingly use this with various purposes. Jeon et al.^5^ showed that the 160‐mm NVA (one‐shot volume scan) mode increased streak artifacts in the skull vertex and posterior fossa compared with the wide‐volume scan (80‐row axial volumetric acquisition with three or four contiguous sections automatically stitched together) mode; however, at the same time, the advantage of 160‐mm NVA mode is its high speed, which is essential for noncooperative children in clinical practice. We deduced that new reconstruction algorithms, such as deep learning‑based image quality improvement techniques, will address the artifact problems, such as streak of 160‐mm NVA mode, in the near future.^29^, ^30^

Regarding the assessment of iodine contrast uniformity in the CT number along longitudinal beam locations, 320‐row ADCT scanner showed remarkable contrast enhancement gradient along the long axis of the patient. Generally, because low‐energy increases x‐ray absorption of iodine by facilitating photoelectric interactions compared with the Compton scattering effects, a low‐tube‐voltage setting, such as 80 kVp, would increase the enhancement of iodine contrast medium more than that with 100 or 120 kVp^31^, ^32^; however, contrast enhancement tending toward the cathode side of the x‐ray tube was less recognized in 256‐row ADCT scanner than in 320‐row ADCT scanner. The trend of iodine contrast enhancement along the long axis of the patient may affect the accuracy of the detection of an ischemic disease and a tumor in brain regardless of the performance of radiologists and computer‐aided diagnosis systems, therefore, the correction of CT numbers is important to keep the longitudinal image uniformity; we deduced that CT numbers in 256‐row ADCT scanner was adjusted by advanced mathematical methods rather than 320‐row ADCT scanner.

X‐ray beam assessment is very important to accurately estimate critical organ dose. However, previous reports related to patient dose estimation in MDCT scanners based on the use of computer simulations failed to consider the heel effect.^33^, ^34^ The heel effect in MDCT scanners with a narrow x‐ray beam is relatively small along the long axis of the patient. We therefore concluded that the heel effect may be negligible in estimating the organ‐absorbed dose in MDCT scanners. In contrast, ADCT scanners with a large x‐ray cone angle have a conspicuous angular distribution of x‐ray beam intensity (energy and dose) along the longitudinal direction. The maximum effective energy difference in the 160‐mm nominal beam width in modern ADCT scanners is approximately 4 keV. Furthermore, the radiation dose on one side of the off‐center position showed a decrease of approximately 40%–45% compared with that in the isocenter position. As the use of a wide x‐ray beam fosters the angular dependence of the emitted spectrum of the x‐ray tube, assessment of x‐ray beam properties along the long axis of the patient in the 160‐mm NVA mode of respective ADCT scanners is important to accurately estimate critical organ dose which shows high radiation sensitivity during CT examinations using computer simulations.

Our study has several potential limitations. Firstly, our study did not verify the moving speed of the CTDP probe for dose profile measurements. Although a significant speed error may have affected the accuracy of dose profile measurements, the results presented are consistent with those reported in previous studies.^2^, ^35^ Secondly, our study did not assess all combinations of tube voltage and bowtie filter settings in respective ADCT scanners. Furthermore, the beam quality properties during ADCT scanning in the 160‐mm NVA mode cannot be determined using only the longitudinal beam properties. Thus, as our presented data alone cannot be used to calculate the radiation dose administered to the critical organs, such eye lens; future studies should investigate the beam properties of in‐plane (*xy*) and longitudinal (*z*) directions for all possible acquisition conditions.

## 5 CONCLUSION

We have described the properties of 256‐ and 320‐row ADCT scanners with 160‐mm wide x‐ray beam in the volume‐acquisition mode. Maximum radiation dose on the anode side of the x‐ray tube showed approximately 40%–45% reduction compared with that on the isocenter position in the ADCT scanners with 160‐mm x‐ray beams; the heel effect properties longitudinally differed throughout the x‐ray beam, and the decrease in the radiation dose in 256‐ and 320‐row ADCT scanners was observed on the patient table side and gantry side respectively.

## CONFLICT OF INTEREST

The authors declare no conflict of interest.
